# Dropping anchor: attachment of peptidylarginine deiminase *via* A-LPS to secreted outer membrane vesicles of *Porphyromonas gingivalis*

**DOI:** 10.1038/s41598-018-27223-5

**Published:** 2018-06-12

**Authors:** Giorgio Gabarrini, Rick Heida, Nienke van Ieperen, Mike A. Curtis, Arie Jan van Winkelhoff, Jan Maarten van Dijl

**Affiliations:** 1 University of Groningen, University Medical Center Groningen, Center for Dentistry and Oral Hygiene, Antonius Deusinglaan 1, 9713 AV Groningen, The Netherlands; 2 University of Groningen, University Medical Center Groningen, Department of Medical Microbiology, Hanzeplein 1, P.O. box 30001, 9700 RB Groningen, The Netherlands; 3 Dental Institute, King’s College London, Guy’s Hospital Tower Wing, SE1 9RT London, United Kingdom

## Abstract

The periodontal pathogen *Porphyromonas gingivalis* has been invoked in the autoimmune disease rheumatoid arthritis (RA). This association relates to the peptidylarginine deiminase of *P*. *gingivalis* (PPAD), an enzyme capable of citrullinating human proteins and potentially contributing to loss of tolerance to citrullinated proteins in RA. PPAD is both retained in the outer membrane (OM) of *P*. *gingivalis* cells and secreted into the extracellular milieu, where it is detected in a soluble form and in association with outer membrane vesicles (OMVs). Recent studies showed that certain *P*. *gingivalis* proteins are retained in the OM through modification with an A-type lipopolysaccharide (A-LPS). Here, we investigated the possible involvement of A-LPS modification in the association of PPAD to the OM and OMVs. The results indicate that the OM- and OMV-associated PPAD is A-LPS-modified. The modified PPAD species is of low abundance in particular clinical isolates of *P*. *gingivalis*, which is not due to defects in the overall synthesis of A-LPS-modified proteins but, rather, to particular traits of the respective PPAD proteins. Lastly, we show that OMV association protects the A-LPS-modified PPAD from proteolytic degradation. Altogether, our observations show that A-LPS modification contributes to OM(V) sorting and ‘protective secretion’ of PPAD.

## Introduction

*Porphyromonas gingivalis* is an anaerobic Gram-negative bacterium and keystone oral pathogen^1,2^ that is mostly known for its role as a causative agent of the inflammatory disease periodontitis^3^. In recent years, this bacterium has garnered interest for its alleged involvement in the etiopathogenesis of the autoimmune disease rheumatoid arthritis (RA)^4–9^. A symptom and possible cause for the disease RA is the loss of tolerance to citrullinated host proteins^7,10–12^. Accordingly, the discovery of a peptidylarginine deiminase in *P*. *gingivalis* (PPAD) was a turning point in the study of the etiology of RA, because this enzyme is capable of citrullinating certain host proteins. In turn, this might cause the production of anti-citrullinated protein autoantibodies (ACPAs), which are highly specific for RA^13,14^. This notion, coupled with the knowledge that periodontitis is clinically associated with RA, suggests a role for PPAD and therefore *P*. *gingivalis* in the etiology of rheumatoid arthritis and highlights the importance of studies on PPAD^7,10^. Specifically, understanding the localization of PPAD would ease the search for its targets and cofactors, rendering the sorting a paramount tool to further the knowledge of the molecular role of this virulence factor in the origin of RA.

**Figure 1:**
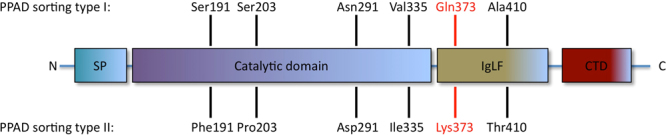
PPAD sorting types are defined by an amino acid substitution at position 373. The PPAD protein of *P*. *gingivalis* comprises four domains, from N- to C-terminal end: the signal peptide (SP), the catalytic domain, the Ig-like fold (IgLF), and the C-terminal domain (CTD) as previously defined^34^. PPAD sorting type I isolates possess a Gln residue at position 373, while sorting type II isolates display a Lys residue in that same position^20^. The Gln and Lys residues at position 373 are marked in red. Also indicated are other substitutions that do not invariably occur in PPAD proteins from sorting type I or II isolates.

Interestingly, three topological species of PPAD can be distinguished: a form bound to the outer membrane (OM)^15–17^, a form bound to secreted outer membrane vesicles (OMVs)^18^ and a secreted form that is present in a soluble state^17,19–21^. This relates to the particular features of the type IX secretion system, which is responsible for the secretion of PPAD and ~30 other OM proteins possessing the same recognition sequence, referred to as ‘C-terminal domain’ (CTD)^22–24^. During export these proteins are subject to post-translational modifications, including attachment to the OM *via* an A-lipopolysaccharide (A-LPS) anchor^15,23,25,26^. Although this has not yet been shown specifically, a modification with A-LPS would also explain the binding of PPAD to the OM and its association with OMVs. In this respect, it should be noted that OMVs are nanostructures resulting from outer membrane ‘blebbing’. Such OMVs are generally used by Gram-negative bacteria to secrete proteins, or to shuttle their cargo to cells or tissues of the host for infection purposes^2,18^. PPAD, in fact, has been reported to be present in OMVs together with several other virulence factors of *P*. *gingivalis*^18,20^. Of note, our recent studies indicate that two PPAD sorting types can be distinguished amongst clinical isolates of *P*. *gingivalis*^20^. Sorting type I isolates produce a dominant OMV-associated PPAD species and relatively lower amounts of the soluble secreted PPAD. In contrast, sorting type II isolates produce restricted amounts of the OMV-associated form of PPAD and substantially higher amounts of the soluble secreted form of PPAD^20^. This difference does not relate to differences in the amounts of PPAD or OMVs produced but, instead, it is correlated with the presence of an amino acid substitution at position 373 in the PPAD amino acid sequence. PPAD of sorting type I isolates has a Gln residue at position 373, whereas PPAD of sorting type II isolates has a Lys residue at this position^20^ (Fig. 1).

In view of the likely importance of A-LPS-modification in the subcellular localization of OM- and OMV-associated proteins, and the importance of PPAD as a virulence factor, the present study was aimed at assessing the extent to which PPAD of *P*. *gingivalis* is A-LPS-modified.

## Materials and Methods

### Bacterial strains and culture conditions

16 *P*. *gingivalis* isolates were obtained from patients with a periodontal diagnosis (Table S1). Additionally, the study included two *P*. *gingivalis* type strains (W83 and ATCC 33277). For comparison, a clinical *Escherichia coli* isolate was included (Table S1). *P*. *gingivalis* strains were grown anaerobically in Brain Heart Infusion (BHI) broth as previously described^27^; cultures were inoculated with 4 days old colonies on blood agar plates. The *E*. *coli* strain was cultured aerobically in Lysogeny Broth (LB) at 37 °C and with shaking (250 rpm), or on LB agar at 37 °C.

### Ethics statement

The bacterial samples used in the present analyses were obtained in a previous study upon written informed consent^4–8^. This previous study received Institutional Review Board approval from the Medical Ethics Committee of the University Medical Center Groningen (METc UMCG 2011/010). It was performed in accordance with the guidelines of the Declaration of Helsinki and the institutional regulations, and all samples were anonymized.

### Immunoblot analyses

To collect secreted proteins of *P*. *gingivalis*, cells were separated from the growth medium by centrifugation and proteins in the growth medium fraction were precipitated with 10% trichloroacetic acid (TCA) as previously described^27^. The collected cells were washed twice with phosphate buffered saline (PBS) to ensure absence of supernatant residues, and frozen at −20 °C. After having been thawed, the cells were resuspended in 300 μL of loading buffer, consisting of lithium dodecyl sulphate (LDS) sample buffer 4× (Life Technologies, Carlsbad, CA, USA), and sample reducing agent 10× (Life Technologies) in MilliQ water. Subsequently, glass beads (Biospec products, Bartlesville, OK, USA) were added to the cells to allow membrane disruption with a Precellys^®^24 Technology tissue homogenizer (Bertin instruments, Montigny-le Bretonneux, France) in three cycles of 30 sec at 6500 rpm with 30 sec breaks in between. Afterward, the glass beads and cellular debris were pelleted by centrifugation at 16100 × *g*, 4 °C for 10 min, and 100 μL of the supernatant were collected and boiled for 10 min at 95 °C. Subsequently, both the proteins from the secreted and the cell fractions were investigated by LDS polyacrylamide gel electrophoresis (PAGE) using NuPAGE gels from Life Technologies^27^.

The isolation of OMVs by ultracentrifugation of growth medium fractions was performed as previously described^20,21^. To disrupt OMVs mechanically, they were exposed to ultrasound in three cycles using a Misonix sonicator S-4000 (amplitude of 80, pulse-on for 15 seconds, pulse-off for 45 seconds, for altogether 3 min of pulse-on time per cycle) prior to precipitation with 10% TCA. To prevent protein degradation, cOmplete Mini protease inhibitors (Roche, Basel, Switzerland) were added immediately prior to sonication, according to manufacturer’s specifications.

Western blotting analysis was performed as described previously^20^, using Amersham™ Protran^®^ 0.45 μm nitrocellulose membranes (GE Healthcare Life Sciences, Little Chalfont, Buckinghamshire, UK) and antibodies specific for either *P*. *gingivalis* A-LPS (mAb1B5)^28^, or PPAD (GP2448)^20^. Membranes were washed with PBS-Tween20 (PBS-T) after overnight incubation with 5% skim milk in PBS, and incubated with primary anti-PPAD or A-LPS antibodies in a 1:10000 dilution. After one-hour incubation, membranes were washed four times for 5 min with PBS-T prior to 45 min incubation with IRDye® 800CW conjugated goat anti-mouse or goat anti-rabbit secondary antibodies (LI-COR Biosciences, Lincoln, NE, USA) at a 1:10000 dilution. When it was necessary to probe a blot with the mAb1B5 monoclonal antibody and a rabbit antibody at the same time, IRDye® 680RD Goat anti-Rabbit IgG (LI-COR) was used as a secondary antibody instead of the IRDye® 800CW conjugated goat anti-rabbit antibody. Subsequently, membranes were washed four times in PBS-T and twice in PBS, before analysis with an Odyssey Infrared Imaging System (LI-COR Biosciences).

### Aqueous two-phase system protein purification

To assess modification of the ~75–85-kDa PPAD species with A-LPS, a protein phase separation assay was applied using the non-ionic detergent Triton X-114 (Sigma-Aldrich, St. Louis, USA)^29^. The cells present in 2.5 OD_600_ units of culture were collected *via* centrifugation at 16100 × *g* 4 °C for 10 min, and resuspended in 300 μL of TEPI buffer, which is composed of TE buffer (Sigma-Aldrich) supplemented with 1 tablet of the cOmplete^TM^ Mini Protease Inhibitor Cocktail (Roche Diagnostics GmbH, Mannheim, Germany) per 10 mL. The growth medium fraction was put aside for later use. The cell fraction was vortexed with glass beads four times for one min, with 1-min intervals on ice, before the supernatant fraction containing soluble cellular and OM proteins was collected *via* centrifugation. The pellets containing glass beads and unbroken cells were washed twice with 300 μL of TEPI buffer and centrifuged. All supernatant fractions with soluble cellular and OM proteins were pooled. Subsequently, 100 μL of Triton X-114 were added to both this pooled fraction with cellular proteins, and to the growth medium fraction. Upon brief vortexing, the Triton X-114-containing samples were incubated for 20 min at 4 °C. Prior to centrifugation at 16100 × *g* and 4 °C for 5 min bromophenol blue (Bio-Rad Laboratories, Richmond, CA, USA) was added to the samples for a better visualization of phase separation in the subsequent steps. Next, the resulting supernatant was overlaid on a 200-μL cushion of 6% (w/v) sucrose (Boom B.V., Meppel, the Netherlands), 10 mM Tris-HCl, pH 7.4, 150 mM NaCl, and 0.06% Triton X-114 to provide a better phase separation. The samples were then heated for 20 min at 37 °C for phase separation, and the separated phases were collected by centrifugation at 400 × *g* for 3 min. To reduce phase contamination, only part of the aqueous phase (800 μL) and part of the detergent phase (90 μL) were collected. The remaining phases were extracted again by adding 90 μL of Triton X-114 to the aqueous phase and 800 μL of TEPI buffer to the detergent phase. Subsequently, proteins in the samples were precipitated with TCA (10% final concentration) by incubation on ice for 30 min. Afterward, the precipitated proteins were collected by centrifugation at 16100 × *g*, 4 °C for 10 min, washed twice with 500 μL of ice-cold acetone, dried at 60 °C for 10 min, separated by LDS-PAGE and analyzed by Western blotting^27^.

### LPS isolation

Isolation and analysis of LPS of *P*. *gingivalis* and *E*. *coli* was performed essentially as described by Geurtsen *et al*.^30^. For each sample, one OD_600_ unit was collected through centrifugation and resuspended in 50 µL of 2× sample buffer^31^ (4% SDS, 20% glycerol, 120 mM Tris-HCl, 0.02% bromophenol blue) before incubation with 50 µL of 1 mg/mL proteinase K for 1 h at 55 °C. Subsequently, the proteinase K was inactivated for 10 min at 95 °C, and samples were diluted ten-fold. 2 µL of the diluted and 2 µL of the undiluted samples were loaded into a Tricine-SDS-polyacrylamide gel. Gels were run at 35 V for the stacking gel part and 105 V for the separation gel part. The separation gel was then fixed overnight in fixing buffer (40% ethanol, 5% acetic acid). Afterward, the gel was incubated, shaking at 40 rpm, in oxidizing buffer (40% ethanol, 5% acetic acid, and 0.7% periodic acid) for 5 min, prior to being washed thrice, in a clean dish, with demineralized water, shaking at 40 rpm, 15 min per wash. Subsequently, the gel was stained with staining reagent (1.33% ammonium hydroxide, 20 mM NaOH, 0.66% silver nitrate (w/v)) for 10 min at 70 rpm. Lastly, the gel was washed thrice with demineralized water in a clean dish for 10 min at 40 rpm prior to incubation at 40 rpm with formaldehyde developer (50 mg citric acid and 0.5 mL 37% formaldehyde per liter) until browning of LPS bands (2–5 min). Development was terminated using 5% acetic acid.

## Results

### Distinction of Triton X-114- and water-soluble forms of PPAD

**Figure 2:**
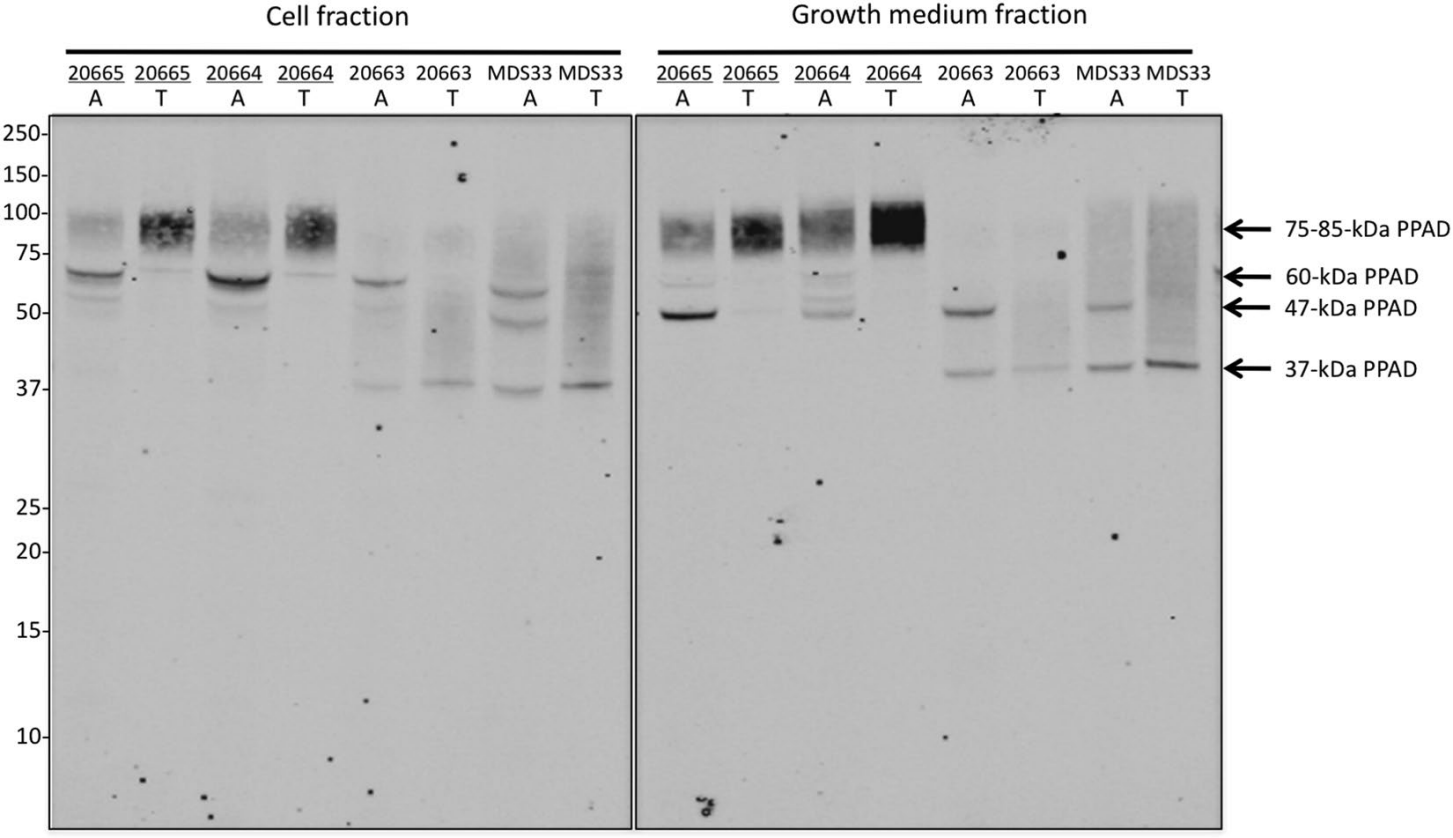
Triton X-114 solubility of different cell-associated and secreted PPAD species. *P*. *gingivalis* isolates were cultured in BHI, and the cell and growth medium fractions were separated by centrifugation. Subsequently, both fractions were extracted with Triton X-114. Upon phase separation at 37 °C, proteins in the different detergent-rich (T) and aqueous (A) phases were analyzed by LDS-PAGE and Western blotting with PPAD-specific antibodies. Names of PPAD sorting type I isolates are underlined. Molecular weights of marker proteins and different PPAD species are indicated. The full-length blot is presented in Figure S1.

To investigate to what extent PPAD exists in hydrophobic and water-soluble states both within *P*. *gingivalis* cells and the growth medium, we established an approach for two-phase protein separation based on the non-ionic detergent Triton X-114. This approach relies on the principle that mixtures of Triton X-114 and water separate into an aqueous and a detergent-rich phase upon heating above the cloud point of Triton X-114. Accordingly, membrane-associated hydrophobic proteins solubilized in mixtures of Triton X-114 and water will segregate into the detergent phase upon heating, while water-soluble proteins will segregate into the aqueous phase. For validation, this approach was applied to four clinical *P*. *gingivalis* isolates showing extreme PPAD sorting type I and II phenotypes. Upon phase separation, a Western blotting analysis was performed using the polyclonal PPAD-specific antibody GP2448. As shown in Fig. 2, this revealed distinct fractionation profiles for PPAD produced by sorting type I and II isolates. Firstly, a thick ~75–85-kDa band, that according to literature data^17^ represents the OM-bound PPAD, was present as a dominant species in the Triton X-114 phase of the cell fraction of the sorting type I isolates 20664 and 20665. Small traces of the ~75–85-kDa band could also be found in the aqueous phase of the cell fraction, but this was most likely due to phase contamination. This fractionation pattern is consistent with the previously proposed A-LPS modification of the ~75–85-kDa form of PPAD^15,23^. Further, we have shown previously that this form of PPAD is also detectable in association with secreted OMVs^20^. Consistent with our previous finding, the present analysis showed that the ~75–85-kDa OMV-associated form of PPAD of sorting type I isolates fractionated with the Triton X-114 phase of growth medium samples. In contrast, a predominantly cell-associated ~60-kDa form of PPAD as well as a secreted form of ~47-kDa fractionated with the aqueous phase (Fig. 2). This is consistent with the view that the ~60-kDa form represents an unprocessed version of PPAD that still possesses the CTD^20^, while the ~47-kDa form represents the soluble secreted PPAD^17,20^.

For the clinical *P*. *gingivalis* sorting type II isolates MDS33 and 20663, a completely different PPAD fractionation pattern was observed. The cell and growth medium fractions of both isolates completely lacked the ~75–85-kDa form of PPAD (Fig. 2). Like the sorting type I isolates, cells of both sorting type II isolates contained the water-soluble ~60-kDa form of PPAD, which was absent from the growth medium. Further, both sorting type II isolates produced the ~47-kDa secreted form of PPAD, similar to the type I isolates. However, in contrast to the sorting type I isolates, both sorting type II isolates produced a ~37-kDa form of PPAD that was detectable both in the cell and growth medium fractions. This ~37-kDa is too small in size to represent an intact form of PPAD. Intriguingly, upon two-phase protein separation, this ~37-kDa form of PPAD did not clearly segregate between the Triton X-114 and the aqueous fractions.

### A-LPS anchors PPAD to the outer membrane

**Figure 3:**
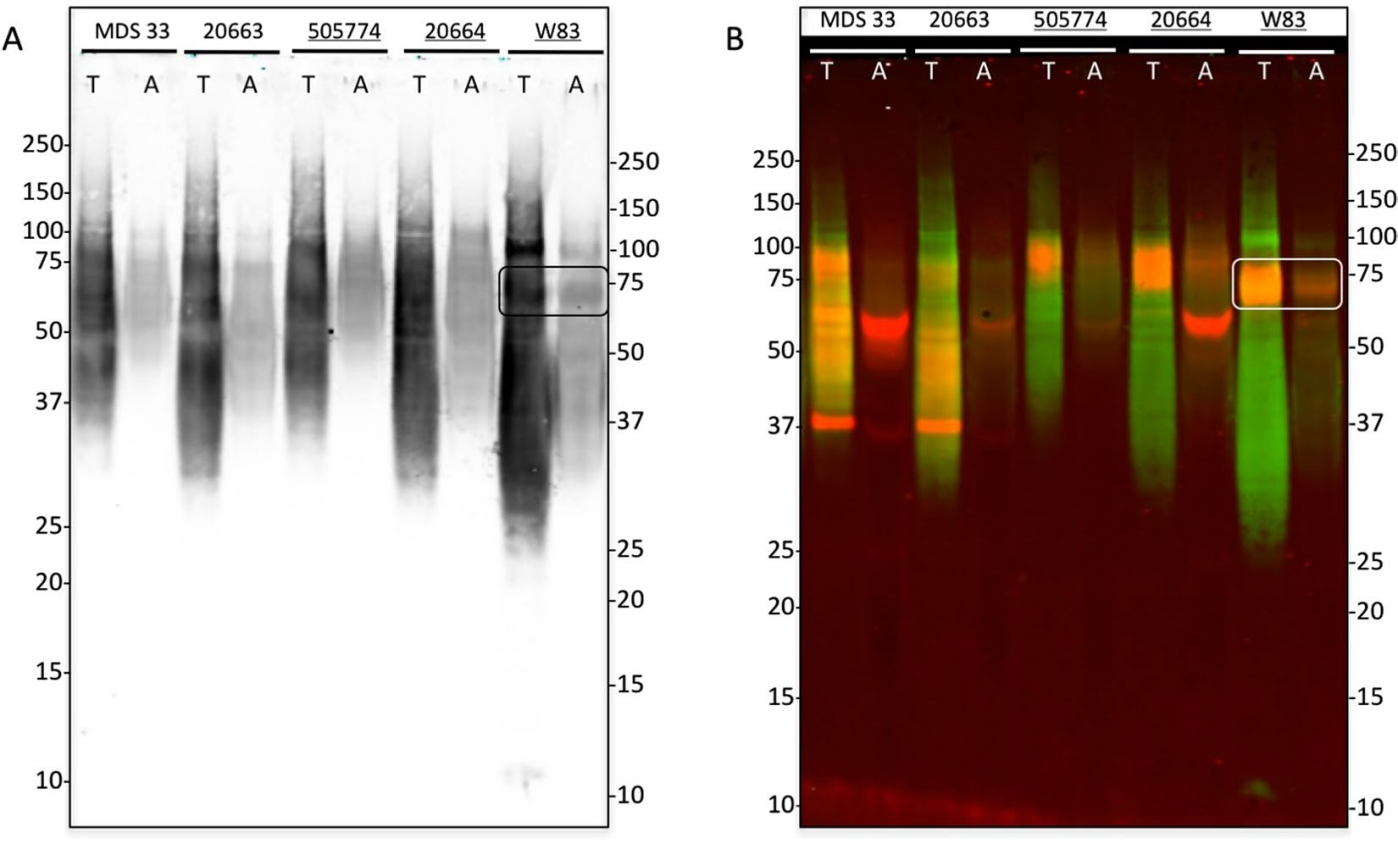
A-LPS modification of PPAD in sorting type I and II isolates of *P*. *gingivalis*. Cells of *P*. *gingivalis* sorting type I and II isolates were cultured in BHI and, subsequently, extracted with Triton X-114. Upon phase separation at 37 °C, proteins in the detergent-rich (T) and aqueous (A) phases were analyzed by LDS-PAGE and Western blotting. In panel A immunodetection was performed with an A-LPS-specific monoclonal antibody. Panel B shows a dual-labeling image upon immunodetection with the A-LPS-specific monoclonal antibody (green signal) and a PPAD-specific rabbit antibody (red signal). Bands representing the A-LPS-modified PPAD species of ~75–85-kDa PPAD are boxed in both panels. Names of sorting type I isolates are underlined. Molecular weights of marker proteins are indicated. The full-length blot is presented in Figure S1. Please note that the order of ‘A’- and ‘T’-labeled lanes is inversed compared to Fig. 2 as samples were loaded in a different order.

To verify that A-LPS is the anchor that binds PPAD to the OM, we performed a Western blotting analysis on the cell fractions of *P*. *gingivalis* isolates of sorting type I and II divided in two phases by two-phase protein separation with Triton X-114. The presence of A-LPS was visualized using the A-LPS-specific monoclonal antibody mAb1B5^28,32^. As shown in Fig. 3, the A-LPS-modified proteins were detected as a very broad pattern of closely migrating bands, rather than distinct individual bands. This was to be expected considering that ~30 different proteins of various sizes appear to be anchored to the OM *via* A-LPS^15^. Furthermore, the bulk of the A-LPS-specific signals were detected in the Triton X-114-rich phase, consistent with the notion that lipidated molecules of *P*. *gingivalis*, such as A-LPS-modified proteins, should fraction in the detergent-phase. Significantly lower amounts of A-LPS-modified proteins were detected in the aqueous phase, most likely as a result of phase contamination. Of note, the overall fractionation profile of A-LPS-modified proteins was independent of the PPAD sorting type of the investigated *P*. *gingivalis* isolates. Further, a thick A-LPS-containing band of ~75-kDa was observed in samples of the *P*. *gingivalis* strain W83, which belongs to the PPAD sorting type I (Fig. 3A, marked by a box). Since this could potentially represent the A-LPS-modified species of PPAD, we repeated the analysis using both the A-LPS-specific mAb1B5 monoclonal and the PPAD-specific GP2448 rabbit antibody, the binding of which can be distinguished with differently labeled secondary antibodies. Accordingly, the A-LPS-specific signal in Fig. 3B is represented in green and the PPAD-specific signal in red, while overlaps between the two signals are represented in yellow-orange. Indeed, the A-LPS-specific green signal was predominantly detected in the detergent-rich phase, with a clear yellow-orange signal marking the position of the ~75–85-kDa band of PPAD in samples of the PPAD sorting type I strains 505774, 20664 and W83. Additionally, the cell-associated ~60-kDa PPAD species was clearly detectable as a red band in the aqueous phase, especially in the case of the sorting type I strain 20664 and the sorting type II strains MDS33. The ~37-kDa PPAD species, instead, was detected as a red band in samples of the PPAD sorting type II isolates, where it was present predominantly in the detergent-rich phase but also in the aqueous phase (Fig. 3B). These findings indicate that the PPAD molecules in the ~75–85-kDa band are A-LPS-modified, while the PPAD molecules in the ~60-kDa and ~37-kDa bands contain little, if any A-LPS.

### PPAD sorting in sorting type II isolates

**Figure 4:**
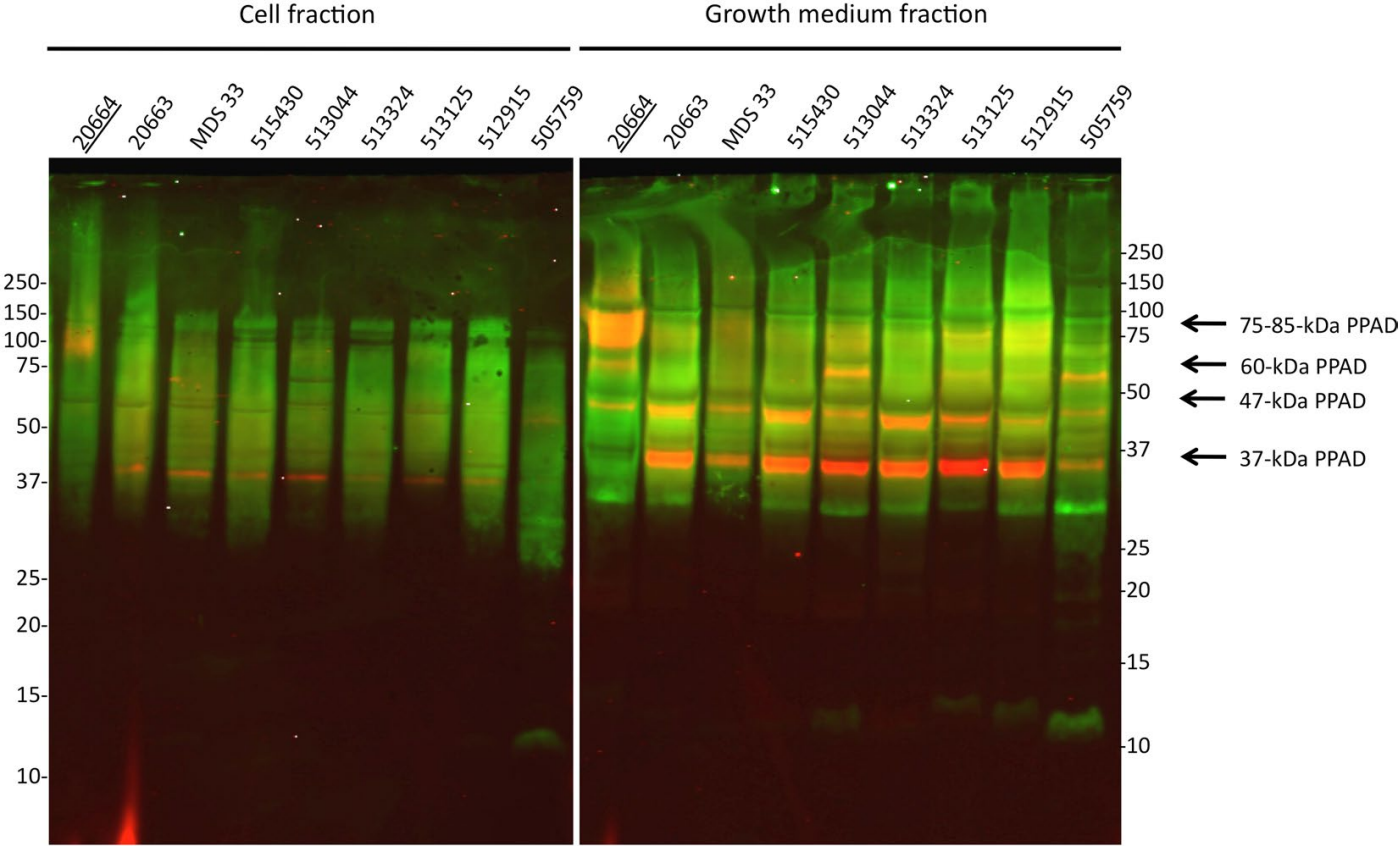
Detection of A-LPS-modified proteins in PPAD sorting type II isolates. *P*. *gingivalis* was cultured in BHI and, subsequently, the cells and growth medium fractions were separated by centrifugation. Proteins in both fractions were separated by LDS-PAGE and analyzed by Western blotting using an A-LPS-specific monoclonal antibody (green signal) and a PPAD-specific antibody (red signal) as indicated for Fig. 3. The name of the PPAD sorting type I isolate used as a control is underlined. Molecular weights of marker proteins are indicated. The full-length blot is presented in Figure S1.

We have previously shown that sorting type II *P*. *gingivalis* isolates secreted very low levels of the ~75–85-kDa OMV-associated PPAD species compared to sorting type I strains^20^. Since the ~75–85-kDa species of PPAD seems to be A-LPS-modified, it was of interest to investigate whether the sorting type II isolates of *P*. *gingivalis* do produce similar levels of A-LPS-modified proteins as the sorting type I isolates. This is indeed the case for both the cell-associated and the secreted proteins of eight sorting type II isolates, as was shown by Western blotting with the A-LPS-specific antibody (Fig. 4; potentially A-LPS-modified proteins are represented in green). Of note, simultaneous immunodetection of PPAD indicates that the dominant PPAD species in the growth medium of these isolates was the unmodified ~37-kDa species (represented as a red band in Fig. 4). Of note, the secreted ~47-kDa species has a yellow-orange appearance in Fig. 4. This most likely relates to its relatively low abundance in the respective molecular weight range of A-LPS-modified proteins. Consistent with the results presented in Fig. 3B, the ~75–85-kDa OM- and OMV-bound PPAD species were displayed significantly only by the sorting type I control isolate 20664 in the cell and growth medium fractions, respectively.

**Figure 5:**
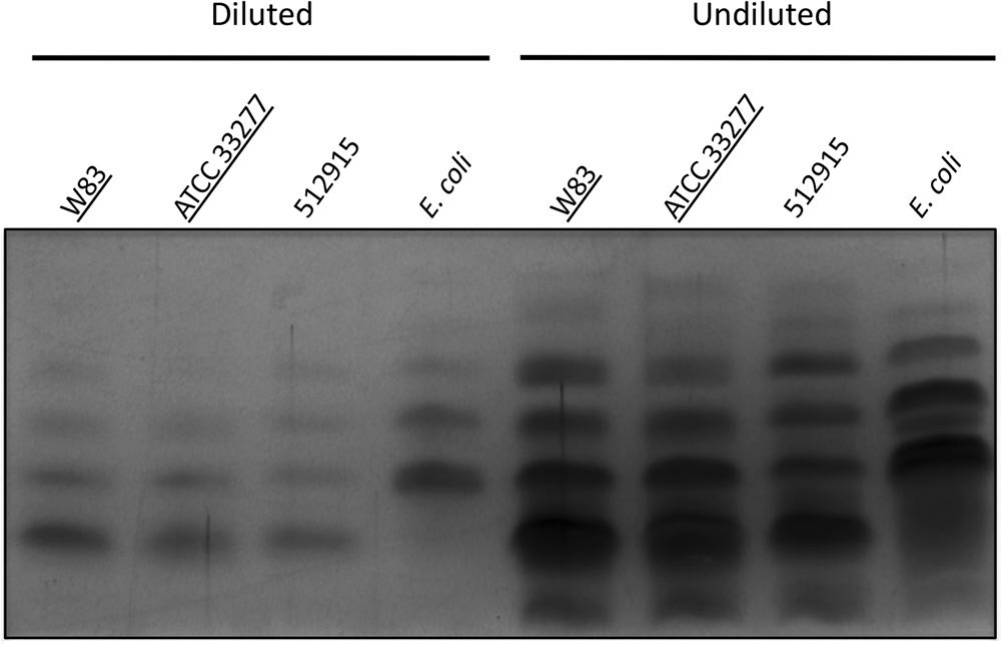
LPS fingerprints of PPAD sorting type I and II isolates. Total LPS was extracted from *P*. *gingivalis* sorting type I and II isolates, and an *E*. *coli* control. Subsequently, the extracted LPS was separated by SDS-PAGE and visualized by silver staining. To detect possible differences in the fingerprints, extracted LPS was loaded in a ten-fold dilution and undiluted. Names of sorting type I isolates are underlined. The full-length gel picture is presented in Figure S1.

To exclude any possible differences in the LPS produced by PPAD sorting type I and II isolates, the total LPS of the sorting type II isolate 512915 was compared to that of the *P*. *gingivalis* type strains W83 and ATCC 3277, which represent the PPAD sorting type I. No differences in the LPS species of these three *P*. *gingivalis* isolates were detectable by SDS-PAGE and subsequent silver-staining (Fig. 5). In contrast, the LPS extracted from the three *P*. *gingivalis* isolates differed substantially from that of an *E*. *coli* control isolate. Together, these findings show that both the PPAD sorting type I and II isolates produce similar amounts of LPS in general and A-LPS-modified proteins in particular.

### Intact OMVs protect A-LPS-modified PPAD against proteolysis

**Figure 6:**
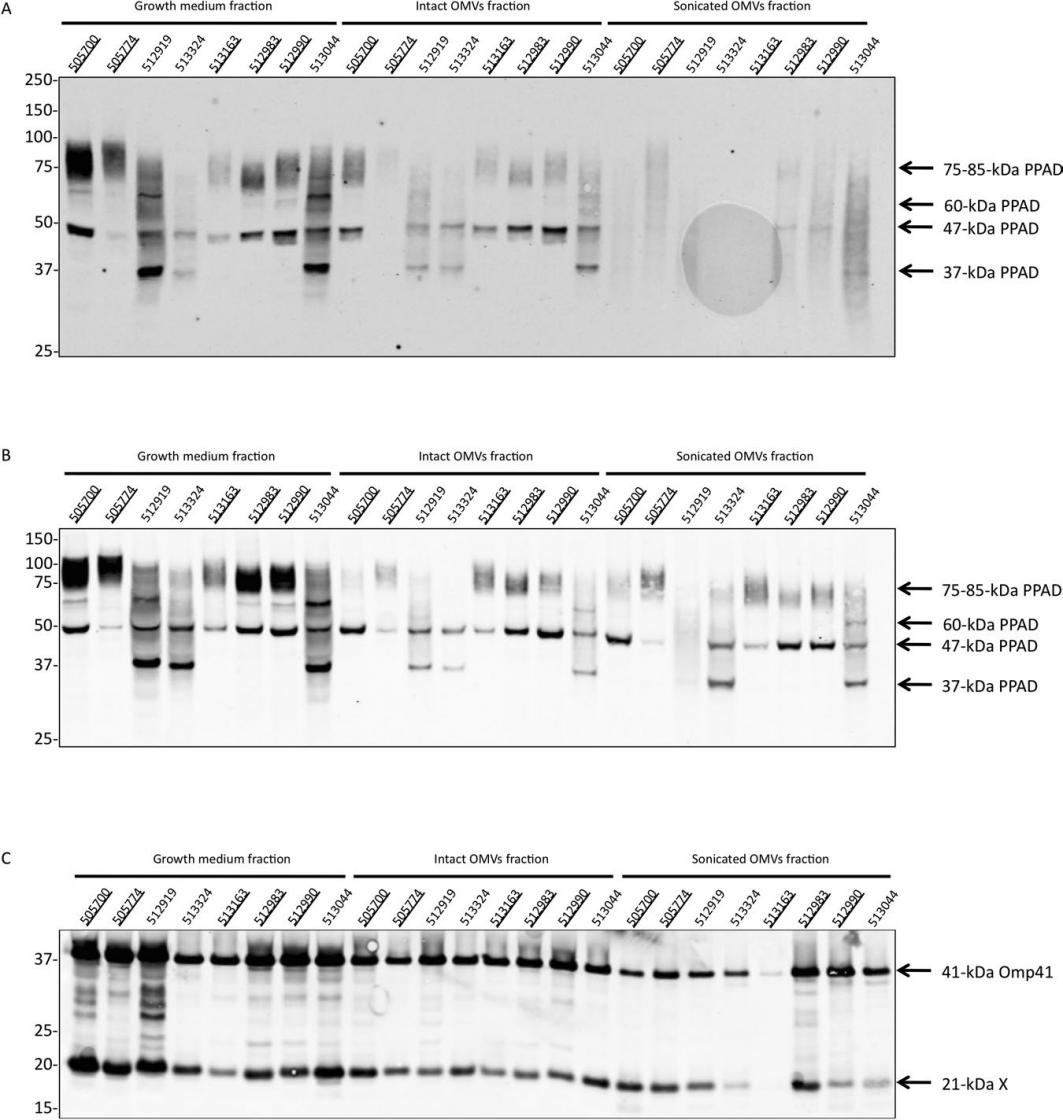
Protective secretion of OMV-associated A-LPS-modified PPAD. *P*. *gingivalis* was cultured in BHI and, subsequently, the cells and growth medium fractions were separated by centrifugation. Next, OMVs were collected from the growth medium fraction by ultracentrifugation, and part of the collected OMV fraction was subjected to sonication either in the absence (**A**,**C**) or presence (**B**) of protease inhibitors. Proteins from the growth medium fraction, intact OMVs and sonicated OMVs were separated by LDS-PAGE and analyzed by Western blotting using a PPAD-specific antibody (**A**,**B**) or an Omp41-specific antibody (**C**). Names of sorting type I isolates are underlined, and molecular weights of marker proteins are indicated. The full-length blots are presented in Figure S1. The band of 21 kDa labeled X represents an unidentified protein that cross-reacts with the Omp41-specific antibody, possibly a degradation product of Omp41.

To investigate whether the OMV-associated A-LPS-modified PPAD can be liberated from the OMVs by mechanical disruption of the OMVs, we subjected the OMVs of different PPAD sorting type I and II isolates to sonication. In particular, OMVs were isolated from the growth medium by ultracentrifugation, exposed to three cycles of sonication, and the resulting fractions were analyzed by LDS-PAGE and Western blotting with PPAD-specific antibodies. Surprisingly, the results shown in Fig. 6A revealed that the PPAD had largely disappeared from the sonicated OMV fraction. To determine whether this was due to degradation by proteases secreted by *P*. *gingivalis*, the experiment was repeated in the presence of a protease inhibitor cocktail. Indeed, the addition of protease inhibitors precluded PPAD degradation during sonication (Fig. 6B), showing that secreted proteases of *P*. *gingivalis* were capable of degrading A-LPS-modified PPAD when the OMV integrity was violated by ultrasound. To assess whether the protection by OMVs was specific for PPAD, the samples from Fig. 6A were also analyzed by Western blotting with antibodies specific for the known OMV cargo protein Omp41^20^. As shown in Fig. 6C, for most investigated isolates, relatively little degradation of Omp41 was detectable upon sonication, even in the absence of protease inhibitors. In the case of isolates 513324 and 513163, also substantial degradation of Omp41 was observed upon OMV sonication, suggesting that these isolates are either more proteolytic than other isolates or that the respective Omp41 proteins are more susceptible to proteolysis than those of other isolates. Altogether, these observations imply that A-LPS-modified PPAD specifically requires the OMV environment for ‘protective secretion’ into the highly proteolytic extracellular milieu of *P*. *gingivalis*.

## Discussion

Based on PPAD’s status as a *P*. *gingivalis* virulence factor and its potential as a key factor bridging periodontitis and the etiopathogenesis of RA, we investigated its attachment to the OM and OMVs of *P*. *gingivalis*. Previous studies have shown that A-LPS is the anchor for proteins secreted by the type IX secretion system. PPAD is also one of the proteins secreted *via* the type IX system and the present study was, therefore, aimed at verifying the A-LPS-modification of PPAD attached to the OM and OMVs.

Our results show the presence of different PPAD species that are differentially sorted in *P*. *gingivalis*. Firstly, a soluble, secreted, ~47-kDa species of PPAD^17,20^ was detected in both sorting type I and II isolates in comparable amounts and not attached to lipids. Interestingly, an alleged A-LPS-modified OM-bound and OMV-bound ~75–85-kDa PPAD species^17,20^ could be detected in PPAD sorting type I isolates and, in severely reduced amounts, also in PPAD sorting type II isolates. Notably, the band representing this species was most prominently detected in the growth medium fraction compared to the cell fraction, which appears to relate to a higher A-LPS density in the OMVs, compared to the outer membrane of the cell^33^. A PPAD species of ~60-kDa was displayed by both sorting type I and II isolates. This PPAD species fractionated with the aqueous phase of cells treated with the detergent Triton X-114, which suggests that it could be localized to the periplasm of *P*. *gingivalis*. In turn, this would imply that the ~60-kDa species represents an unprocessed, uncleaved form of PPAD, as was suspected based on its size and solubility^20,21^. Lastly, a ~37-kDa PPAD species was observed only in sorting type II isolates. Upon Triton X-114 extraction, this ~37-kDa form of PPAD did not clearly segregate between the detergent and the aqueous fractions. This suggests that the ~37-kDa form represents two similarly-sized degradation products of PPAD with different hydrophobicity. For example, the hydrophobic form of the ~37-kDa that could represent a derivative of the ~75–85-kDa species. In this respect, it is noteworthy that the PPAD sorting type II phenotype is associated with a Gln to Lys substitution at residue 373, which is exposed to the surface of PPAD^20^. The presence of this Lys residue at the membrane surface could make PPAD proteins with the sorting type II susceptible to cleavage by the secreted lysine-specific gingipain (Kgp) of *P*. *gingivalis*, which might explain the presence of the ~37 kDa PPAD species.

To verify whether PPAD binding to the OM and OMVs involves an A-LPS anchor, we made use of the mAb1B5 antibody^28,32^, which was raised specifically against *P*. *gingivalis* A-LPS. Upon probing with both mAb1B5 and a PPAD antibody, we discovered that a strong A-LPS signal at around 75-kDa aligns with the PPAD signal at this position in the Western blot. This supports the view that the ~75–85-kDa PPAD species fractionating with the detergent Triton X-114 is A-LPS-modified. Conversely, Western blotting with mAb1B5 indicates that the soluble ~60-kDa form of PPAD does not contain A-LPS, consistent with the periplasmic localization as proposed above. Together, these data allow us to correlate the differentially stained A-LPS and PPAD bands in Figs 3B and 4, in particular because the 75–85 kDa species of PPAD is nearly completely absent from the PPAD sorting type II cells and growth medium fractions. Importantly, the Western blotting analysis with mAb1B5 demonstrated the presence of comparable amounts of potentially A-LPS-modified proteins in all investigated *P*. *gingivalis* isolates, regardless of their PPAD sorting type. This shows that the PPAD sorting types I and II are not distinguished by differences in the overall A-LPS modification of proteins. Further proof of this view was obtained by the analysis of total LPS extracted from cells with different PPAD sorting types, which showed no detectable differences.

Lastly, by subjecting OMVs of either PPAD sorting type to sonication, the A-LPS-modified PPAD became susceptible to proteolysis. On this basis, we conclude that the A-LPS-modification serves not only to localize PPAD to OMVs but also to place it in a position where it is protected from proteolysis by the highly active secreted proteases of *P*. *gingivalis*, including arginine- and lysine-specific gingipains. Whether this means that the OMV association segregates the A-LPS-modified PPAD physically from the secreted proteases, or whether particular protease cleavage sites are protected by the OMVs remains to be determined. This is a relevant question for future studies, because a previous study has shown that gingipains are also detectable as cargo of OMVs from *P*. *gingivalis* strain W50^18^. Of note, these previous results indicated that the gingipains were predominantly exposed on the OMV surface, but absent from the OMV lumen^18^.

Taken together, the present findings correlate A-LPS-modification of PPAD with the anchoring of this protein to the OM and OMVs of *P*. *gingivalis*. Additionally, they show that there are no differences in the overall A-LPS synthesis or overall A-LPS modification of exported proteins in PPAD sorting type I and II isolates. This supports the notion that Gln373 of PPAD is critical for A-LPS modification, and that the Q373K substitution encountered in the PPAD of sorting type II isolates hinders the A-LPS modification of this important virulence factor. It is currently not known whether the difference in A-LPS modification and PPAD sorting observed in the type I and II *P*. *gingivalis* isolates may have clinical implications for periodontitis and RA. Both PPAD sorting types I and II have been found in at least one periodontitis patient with RA and at least one periodontitis patient without RA. This leads us to believe that the PPAD sorting type is not a decisive, tipping factor for triggering RA. However, considering the fact that RA is a multifactorial disease and that patient data on the analyzed isolates were in most cases incomplete with respect to the diagnosis of RA, it is presently impossible to draw conclusions on possible implications of the PPAD sorting type in relation to RA.

### Ethics

The present research has no particular ethical implications.

## Electronic supplementary material

Supplementary information
