# Supplementary material for: Dropping anchor: attachment of peptidylarginine deiminase via A-LPS to secreted outer membrane vesicles of Porphyromonas gingivalis
Source: Sci Rep. 2018 Jun 12;8:8949. doi: 10.1038/s41598-018-27223-5 (PMC5997701; doi:10.1038/s41598-018-27223-5)
Supplement: Supplementary file 1 — Supplementary information [file 41598_2018_27223_MOESM1_ESM.pdf]

Supplementary information

Dropping anchor: attachment of peptidylarginine deiminase *via* A-LPS to secreted outer membrane vesicles of *Porphyromonas gingivalis*

Giorgio Gabarrini[1,2], Rick Heida[2], Nienke van Ieperen[2], Mike A. Curtis[3], Arie Jan van Winkelhoff[1,2], and Jan Maarten van Dijl[2#]

[1]University of Groningen, University Medical Center Groningen, Center for Dentistry and Oral Hygiene, Antonius Deusinglaan 1, 9713 AV, Groningen, the Netherlands

[2]University of Groningen, University Medical Center Groningen, Department of Medical Microbiology, Hanzeplein 1, P.O. box 30001, 9700 RB, Groningen, the Netherlands

[3]Dental Institute, King's College London, Guy's Hospital Tower Wing, SE1 9RT, London, United Kingdom

Table S1. Panel of isolates - Panel of bacterial isolates used in this study, including species, type of strain and sorting status.

Sample	Species	Strain type	Sorting status
**W83**	*P. gingivalis*	Reference	Type I
**ATCC 33277**	*P. gingivalis*	Reference	Type I
**20664**	*P. gingivalis*	Clinical isolate	Type I
**20665**	*P. gingivalis*	Clinical isolate	Type I
**505700**	*P. gingivalis*	Clinical isolate	Type I
**505774**	*P. gingivalis*	Clinical isolate	Type I
**513163**	*P. gingivalis*	Clinical isolate	Type I
**512983**	*P. gingivalis*	Clinical isolate	Type I
**512990**	*P. gingivalis*	Clinical isolate	Type I
**20663**	*P. gingivalis*	Clinical isolate	Type II
**MDS33**	*P. gingivalis*	Clinical isolate	Type II
**505759**	*P. gingivalis*	Clinical isolate	Type II
**512915**	*P. gingivalis*	Clinical isolate	Type II
**512919**	*P. gingivalis*	Clinical isolate	Type II
**513125**	*P. gingivalis*	Clinical isolate	Type II
**513324**	*P. gingivalis*	Clinical isolate	Type II
**513044**	*P. gingivalis*	Clinical isolate	Type II
**515430**	*P. gingivalis*	Clinical isolate	Type II
**EC001**	*E. coli*	Clinical isolate	/

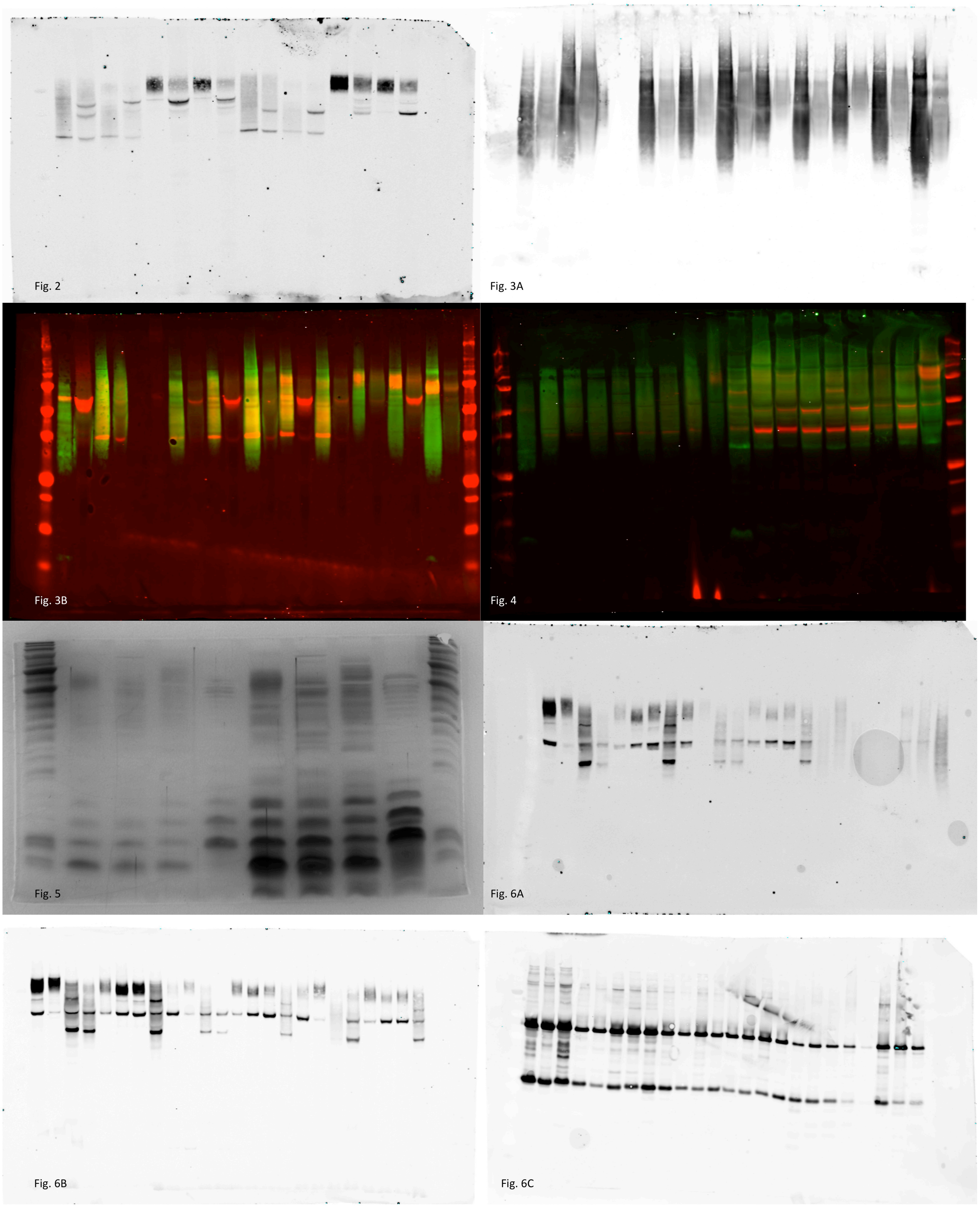

Figure S1. Collection of original images - Panel of the original versions of Figures 2, 3A and 3B, 4, 5, 6A, 6B, and 6C. The figures in the manuscript have been cropped and the order of the samples, when needed, has been reorganized for clarity and ease of presentation.
